# Object detectors involving a NAS-gate convolutional module and capsule attention module

**DOI:** 10.1038/s41598-022-07898-7

**Published:** 2022-03-10

**Authors:** Thanaporn Viriyasaranon, Jang-Hwan Choi

**Affiliations:** Division of Mechanical and Biomedical Engineering, Graduate Program in System Health Science and Engineering, Ewha Womans University, Seoul, 03760 Republic of Korea

**Keywords:** Electrical and electronic engineering, Computer science, Information technology, Software

## Abstract

Several state-of-the-art object detectors have demonstrated outstanding performances by optimizing feature representation through modification of the backbone architecture and exploitation of a feature pyramid. To determine the effectiveness of this approach, we explore the modification of object detectors’ backbone and feature pyramid by utilizing Neural Architecture Search (NAS) and Capsule Network. We introduce two modules, namely, NAS-gate convolutional module and Capsule Attention module. The NAS-gate convolutional module optimizes standard convolution in a backbone network based on differentiable architecture search cooperation with multiple convolution conditions to overcome object scale variation problems. The Capsule Attention module exploits the strong spatial relationship encoding ability of the capsule network to generate a spatial attention mask, which emphasizes important features and suppresses unnecessary features in the feature pyramid, in order to optimize the feature representation and localization capability of the detectors. Experimental results indicate that the NAS-gate convolutional module can alleviate the object scale variation problem and the Capsule Attention network can help to avoid inaccurate localization. Next, we introduce NASGC-CapANet, which incorporates the two modules, i.e., a NAS-gate convolutional module and capsule attention module. Results of comparisons against state-of-the-art object detectors on the MS COCO *val-2017* dataset demonstrate that NASGC-CapANet-based Faster R-CNN significantly outperforms the baseline Faster R-CNN with a ResNet-50 backbone and a ResNet-101 backbone by mAPs of 2.7% and 2.0%, respectively. Furthermore, the NASGC-CapANet-based Cascade R-CNN achieves a box mAP of 43.8% on the MS COCO *test-dev* dataset.

## Introduction

Object detection, a fundamental and challenging task in computer vision that has been widely adopted in real-world applications, aims to localize and classify multiple objects in an image. Typically, deep learning-based object detectors can be divided into two categories based on their architecture: one-stage methods^1–8^ such as YOLO^9^ and SSD^10^, which directly utilize convolutional neural networks (CNNs) to classify and predict the bounding boxes of the object, and two-stage detectors^11–27^ such as Faster R-CNN^28^ that adopt a region proposal network (RPN) to extract the region proposal from the CNN backbone feature map to classify and predict the bounding boxes. Generally, object detection systems in both categories involve three components: a backbone for basic feature extraction, a neck for fusing multi-level features, and a detection head to realize the object classification and bounding box regression. Two-stage object detection systems have an additional component, RPN, to propose candidate object bounding boxes. Owing to the architectural differences between the two categories, the two-stage detectors have high localization and object recognition accuracy, whereas the one-stage detectors achieve high inference speed.

Most backbone networks for detection are generally used for classification, e.g. ResNet^29^ and VGG16^30^, with the last fully connected layers removed. For better detection accuracy, a deeper and densely connected backbone is adopted to replace its shallower and sparsely connected counterpart. However, a classification network usually reduces the spatial resolution of the feature maps with a large downsampling factor, which is beneficial for visual classification, although the low-spatial resolution impedes the accurate localization of large objects and recognition of small objects. There have been several attempts to alleviate the issues arising from scale variation and instances of small objects in object detection, such as proposing new backbone architectures that maintain a high spatial resolution in the deep layers^31–33^, modification of convolution by utilizing Atrous convolution^26^, and adoption of an attention mechanism^34^. These approaches have achieved considerably higher detection performance. Nevertheless, these methods have been based on hand-crafted network design, which requires expert knowledge and experience. To overcome this limitation, the use of neural architecture search (NAS) frameworks, which automatically determine the optimal network architecture for a certain task and dataset, has attracted attention, especially in computer vision tasks including object detection. For example, DetNAS^33^ and Hit-Detectors^35^ have used NAS to search for a new backbone, NAS-FPN^36^ and Auto-FPN^37^ have attempted to search the architecture for the neck (feature fusion network). However, the optimization of the object detector’s backbone based on NAS has difficulty in promptly evaluating the candidate models in the search space. Furthermore, the architecture of the backbone keeps changing during the search, which is computationally infeasible and time-consuming. To reduce the computational cost, we proposed a NAS-gate convolutional module, which optimized only the standard convolution on the classification network backbone by exploiting the NAS gradient search method. We also utilized multiple kernel sizes and dilated rates in the convolutional operation as the candidate operation on the NAS searching operation in order to improve object scale variation detection performance of the designed NAS-based backbone. In other words, adopting the NAS-gate convolution module in the classification network backbone can improve efficiency and overcome issues arising from object scale variation with a smaller computation load compared to previous NAS object detector backbones.

While a better feature extractor certainly plays an important role, considerable improvement comes from better design of work architectures for the feature fusion network or the neck. Feature Pyramid Network (FPN)^36^ is one of the representative model architectures for the feature fusion neck that has achieved remarkable performance in object detection. FPN propagates features in a top-down path, and the low-level features can be improved with stronger semantic information from higher-level features. Furthermore, there have been several works optimizing FPN architecture^36–38^ to improve the feature representation of FPN. However, FPN and FPN-based methods suffer from information loss in the highest-level feature map. Although the information loss can be mitigated by combining the global context feature, this strategy leads to the spatial relationship between objects in the images getting lost. Another effective approach to mitigate information loss and improve the feature representation is utilizing an attention mechanism such as SENet^39^ and CBAM^40^. The attention mechanism improves the feature representation by concentrating only on relevant features and ignoring others. However, previous attention mechanisms have exploited the global context, which results in losing spatial relationships. In this work, we propose an attention mechanism, named capsule attention module, which improves feature representation without losing spatial relationships. Our capsule attention module is based on a capsule network, which can encode spatial information and account for the spatial relationships between the objects in the image. Using the strong spatial relation accounting ability of the capsule network, the capsule attention module can identify stronger relationships between the underlying object than existing attention mechanisms. Therefore, adopting the capsule attention at the highest level of FPN or FPN-based methods can alleviate the information loss problem without losing spatial relationships, improving the localization ability.

We incorporated both proposed modules, i.e., the NAS-gate convolutional module and capsule attention module, into state-of-the-art object detectors such as Faster R-CNN and Cascade R-CNN to create NASGC-CapANet. Experiment results demonstrate that NASGC-CapANet substantially improves the performance of the baseline object detectors. NASGC-CapANet-based Faster R-CNN with FPN increases mAP by 5.8%, and NASGC-CapANet-based Cascade R-CNN with PAFPN increases mAP by 1.0% on MS COCO *test-dev*. The main contributions of our work are summarized as follows:

- We proposed the NAS-gate convolutional module, which utilized the NAS operation based on differentiable architecture search (DARTS) with multiple kernel sizes and dilation rates for the convolutional operation of the classification backbone network to decrease the computation cost of NAS-based backbones and alleviate the issues arising from the object scale variation.
- We introduced a capsule attention module, based on a capsule network, to improve the feature representation by mitigating the information loss problem of FPN using the strong spatial relation ability of the capsule network.
- We evaluated the performance of both the proposed modules and the incorporation of the proposed modules with state-of-the-art object detectors, NASGC-CapANet, on MS COCO and PASCAL VOC. The experiment results show that NASGC-CapANet considerably improves the detection performance compared to start-of-the-art baseline object detectors.

## Method and experiment

**Figure 1:**
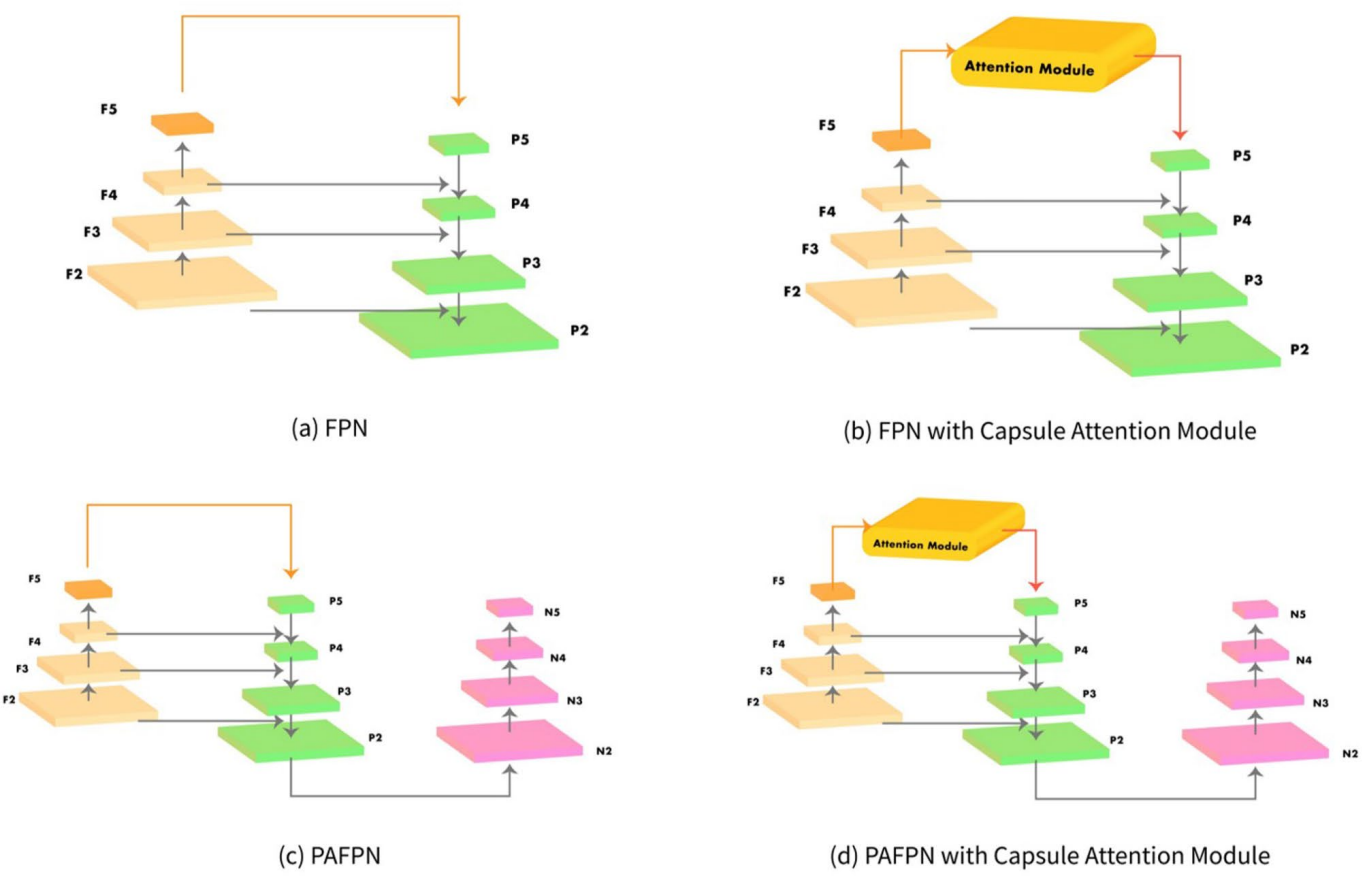
Architecture of feature fusion networks with and without the capsule attention module—(**a**) FPN; (**b**) FPN with capsule attention module; (**c**) PAFPN; (**d**) PAFPN with capsule attention module.

In this section, we describe the architecture design of the proposed NASGC-CapANet, which is a combination of the state-of-the-art object detectors and our proposed modules. In general, the NAS-gate convolutional module and capsule attention module can both be incorporated in one-stage as well as two-stage object detectors. However, most studies in this domain focus on incorporating these modules in two-stage detectors such as Faster R-CNN^28^ and Cascade R-CNN^15^. In order to mitigate the problems arising from object scale variation, we optimized the feature extractor ability of the backbone by replacing the standard convolution of the classification backbone network with the proposed module, i.e., a NAS-gate convolutional module based on Neural Architecture search method, to increase the detection performance on the multiscale objects in the images with smaller computation cost compared to the NAS-based object detectors backbones. In order to enhance the localization ability of the object detectors, we improve the feature representation of the feature fusion network or neck by alleviate the information lost at the highest feature level problem with the capsule attention module. The capsule attention module was designed to incorporate with the feature fusion networks, i.e., FPN and FPN-based methods such as PAFPN from PANet, which the architecture are shown in Fig. 1a, c, respectively. Capsule attention module is adopted at the highest level of the FPN and PAFPN as shown in Fig. 1b, d, respectively.

### NAS-gate convolutional module

The NAS-gate convolutional module was designed to explore the object detector’s backbone architecture by optimizing the standard convolution using a Neural Architecture Search approach. Generally, NAS automatically finds an optimal network architecture for a certain task and dataset. The NAS domain involves three key areas: reinforcement learning-based methods that train a recurrent neural network (RNN) controller to generate the cell structure and form the CNN architecture; evolutionary algorithm (EA)-based methods that update the architecture or network by mutating the current best architectures; and gradient-based methods, which were utilized in the NAS-gate convolutional module, that define an architecture parameter for the continuous relaxation of the search space, thereby allowing differentiable optimization in the architecture search to accelerate the search process. Specifically, the NAS-gate convolutional module uses the NAS gradient-based method, named Differentiable ARchiTecture Search (DARTS)^41^, to search for the optimal condition of the convolutional operation of the object detector’s backbone.

In order to mitigate the computational infeasibility and time consumption of the NAS-based object detector backbone, we utilized the NAS method to search for the optimal convolutional operation of classification network backbones such as ResNet-50 and ResNet-101, instead of searching for new backbone architectures. In the NAS-gate convolutional module, each $3 \times 3$ convolutional operation was defined as the computation cell within which the NAS operation searched for the final backbone architecture. Each cell was regarded as a directed acyclic graph, which was formed by sequentially connecting N nodes. Each node $y^{(i)}$ was a feature representation in convolutional networks, and each directed edge (*i*, *j*) was associated with some operation $p^{(i,j)}$ that transformed $y^{(i)}$. The output of each node was obtained by the summation of the transformed $y^{(i)}$ with operations $p^{(i,j)}$:

1 $$\begin{aligned} {{\hat{y}}}^{(i)} = \sum _{i<j}p^{(i,j)}(y^{(i)}) \end{aligned}$$

**Figure 2:**
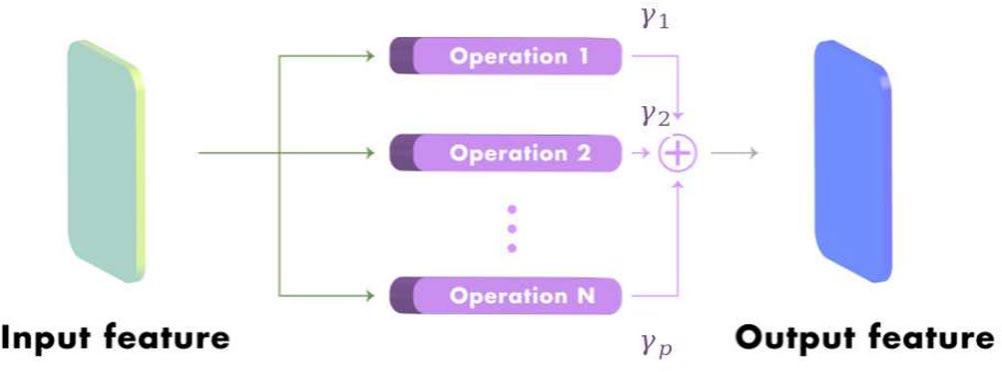
Mixing operation of NAS-gate convolutional module for generating the optimal convolutional operation of the final backbone architecture.

In order to optimize the standard convolution of object detector backbone, we define the node $y^{(i)}$ as the feature representation input of the $3 \times 3$ convolutional operations in ResNet-50 and ResNet-101, *P* is the set of candidate operations where each operation represented function $p(\cdot )$ to be applied to $y^{(i)}$. As the differing scales of objects require different kernel sizes or dilated rates in the convolutional operation to effectively extract features of the scale-variant object in the images, we utilized two different kernel sizes and two different dilated rates as following for the candidate operations in *P*:

- $3\times 3$ dilated convolution with rate 1,
- $3\times 3$ dilated convolution with rate 2,
- $5\times 5$ dilated convolution with rate 1,
- $5\times 5$ dilated convolution with rate 2

To make the search space continuous, the categorical choice of a particular operation was defined as the softmax overall possible operations:

2 $$\begin{aligned} p^{(i,j)}(y) = \sum _{p\in P } \dfrac{exp(\gamma ^p)}{\sum _{p'\in P_b} exp(\gamma ^{p'})} p(y), \end{aligned}$$

where the operation mixing weights for each node *(i,j)* are parameterized by a vector $\gamma ^p$ as shown in Fig. 2. Then, the aim of the architecture search was to learn a set of variables $\gamma$. At the end of the DARTS search, a discrete architecture is obtained by replacing each mixed operation $p^{(i,j)}(y)$ with the most likely operation (i.e., $p(y) = argmax_{p\in P}\gamma ^p$). However, selecting only one operation for each node can lead to a decreased efficiency of feature extraction because a single convolutional operation cannot extract features of scale-variant objects in the images as effectively as the mixing operation, which is a combination of multiple convolutional operation options with weight parameters $\gamma$. The mixing operation can provide the feature representation that contains important features for all scale object sizes via the combination process. Therefore, we did not change the final architecture to discrete architecture; we instead utilized the architecture with a mixing operation during training at the end of the search operation. Furthermore, the learning of the variables $\gamma$ in DARTS was updated through the gradient descent and optimized using the validation loss. However, updating the parameter $\gamma$ by optimizing the validation loss is time-consuming for learning until the optimal architecture is obtained. Therefore, we updated the parameter $\gamma$ by optimizing the training loss, $L_{train}(w,\gamma )$:

3 $$\begin{aligned} L_{train}(w,\gamma ) = L_{cls}(w,\gamma ) + L_{loc}(w,\gamma ), \end{aligned}$$

where the $L_{cls}(w,\gamma )$ represents the loss function for object classification and the $L_{loc}(w,\gamma )$ indicates the loss function for bounding box localization. In addition, the object classification loss and bounding box localization loss were determined by the weights of the network *w* and the operation mixing weights $\gamma$. The algorithm 1 is the NAS-gate convolutional module searching algorithm. 
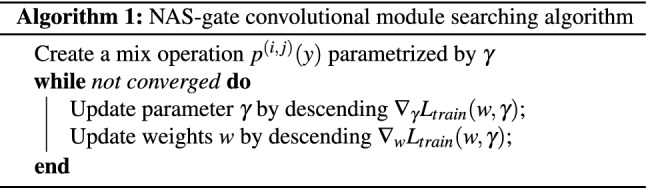


### Capsule attention module

In this subsection, we present the details of our proposed capsule attention module. The capsule attention module has been designed based on the structure of Capsule Network or CapNet^42^. CapNet was proposed to overcome the challenges faced by convolutional neural networks (CNNs), specifically, the loss of information via the pooling process, sensitivity to object orientation, and difficulty in transferring the understanding of the geometric relationship to new viewpoints. Therefore, the concept architecture and optimization process is different from the CNN. The capsule in a CapNet is a group of neurons that utilizes a vector to represent the instantiating parameters of a specific type of entity such as an object or object parts. The length of a capsule vector represents the probability of the objects existing in the image while the direction of the vector represents the corresponding pose information. Therefore, CapNet is more robust to changes in the orientation and size of the input. Furthermore, CapNet can encode spatial information and account for the spatial relations between the parts of the image. Accordingly, we exploited these abilities of CapNets to generate the attention mask, which was applied to improve the feature representation by emphasizing the object-related features and suppressing unrelated ones, in the proposed capsule attention module. In addition, we utilized the capsule attention module in FPN and FPN-based methods in order to increase the performance of the detectors by alleviating the highest-level information loss problem and enhancing the feature representation with strong semantic information, especially the spatial information and spatial relationships between the objects in the images.

**Figure 3:**
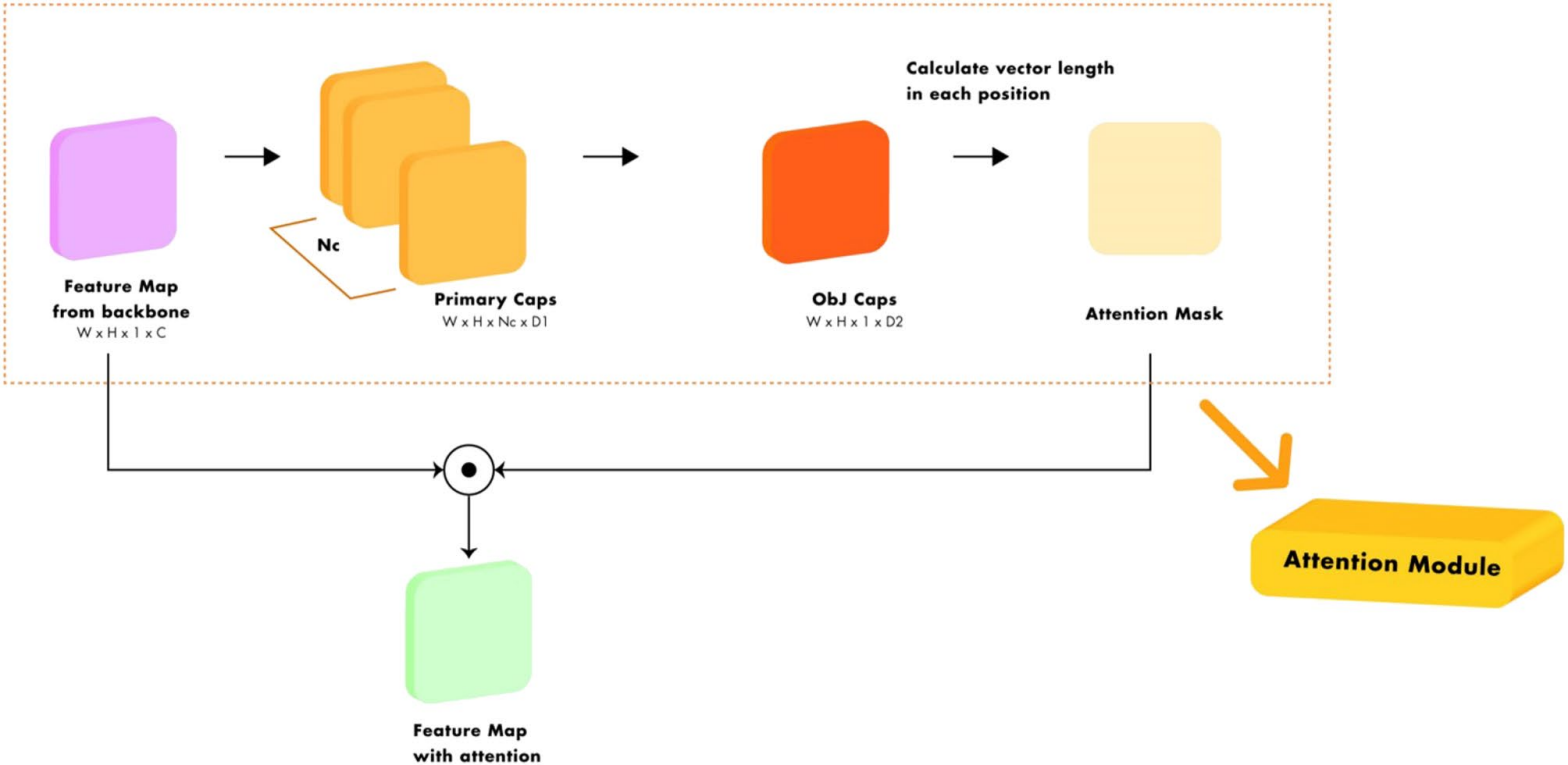
Architecture of the capsule attention module.

The proposed capsule attention module consists of two layers of capsules, as illustrated in Fig. 3. The first layer of capsules, named primary caps, reformulates the input feature representation, which was the feature representation of the highest level of the backbone, into $N_c$ channels of convolutional $D_1$ capsules, $\mathbf{Z }_i$ where $N_c$ is defined as 12 and $D_1$ was 52. Each capsule in primary caps consists of 52 convolutional units with a $3 \times 3$ kernel and a stride of 1. In addition, the output of the primary caps layer has [$N_c\times H \times W$] capsule outputs (each output was a 52-D vector), where *H* and *W* denote the height and width of the input feature representation, respectively. Each capsule in primary caps is transformed to provide a vote with transformation matrix $W_{ij}$. The vote is:

4 $$\begin{aligned} \hat{\mathbf{Z }}_{j|i} = W_{ij}\mathbf{Z }_i \end{aligned}$$

The second layer is the object caps (Obj caps) layer that includes only one $D_2$ capsule with a single channel, where we define $D_2$ as 52. Each capsule in this layer receives the votes from the primary caps as input, and the vector outputs of this layers are computed through dynamic routing^42^. The routing mechanism identifies a coefficient $r_{ij}$ for each vote $\hat{\mathbf{Z }}_{j|i}$, which are all determined by the iterative dynamic routing process, and takes all votes to calculated weighted sum over all votes as output vectors $\mathbf{t }_j$:

5 $$\begin{aligned} \mathbf{t }_j = \sum _{i} r_{ij}\hat{\mathbf{Z }}_{j|i} \end{aligned}$$

The coefficient $r_{ij}$ between capsule *i* and all the capsules in the primary caps are determined by a ”routing softmax” to enforce the probabilistic nature of coefficient $r_{ij}$ to be non-negative number, and their summation equals to one. Furthermore, the routing softmax utilized the log prior probabilities $b_{ij}$, which can be defined as network parameters, and learned at the same time as all the other weights, to determine the coefficient $r_{ij}$:

6 $$\begin{aligned} r_{ij} = \dfrac{exp(b_{ij})}{\sum _{k} exp(b_{ik})} \end{aligned}$$

As the length of the output vector of the capsule represents the probability that objects are presented, the capsule uses a non-linear ”squashing” function to ensure that each feature related to the object is represented by a length slightly less than one while the background feature has a vector length of almost zero. The squashing function is defined as:

7 $$\begin{aligned} \mathbf{v }_j = \dfrac{\left| \left| \mathbf{t }_j\right| \right| }{1+ \left| \left| \mathbf{t }_j\right| \right| ^2}\dfrac{\mathbf{t }_j}{\left| \left| \mathbf{t }_j\right| \right| } \end{aligned}$$

where $\mathbf{v }_j$ is the vector output of capsule *j*.

A final attention mask is created by computing the length of the capsule vectors in the final layer, Obj Caps, and the attention mask is multiplied to the input feature, which is the feature representation of the highest level of the backbone to improve the feature representation.

The capsule attention module is a new concept of the attention mechanism, which is designed to strengthen feature representation power by exploiting global context without losing spatial relation. For object detection, we adopt the capsule attention module at the highest-level of FPN and FPN-based methods in order to improve feature representation and alleviate information loss problem, which results in improving the localization performance of the object.

### Dataset

We evaluated the performance of the proposed NASGC-CapANet on two different benchmark datasets including PASCAL VOC^43^, which is the public dataset for VOC2012 challenges that is available at http://host.robots.ox.ac.uk/pascal/VOC/voc2012/index.html and MS COCO^44^, which is the public dataset for the MS COCO challenge that is available at https://cocodataset.org. Since it is nearly impossible to obtain informed consent for all persons present in the two Internet image datasets, the data were collected without consent. All methods on the data were performed in accordance with relevant guidelines and regulations. In order to remove privacy concerns, we cropped the head area from the image. PASCAL VOC contains 20 object classes. The union of *VOC-2007 trainval* and *VOC-2012 trainval* (10k images) was used for the model training, and *VOC-2007 test* (4.9k images) was used for the model evaluation. The performance on the PASCAL VOC was evaluated using the mAP scores with an intersection over union (IoU) of 0.5. MS COCO 2017 contains 80 object classes with 118k and 5k images for training (*train-2017*) and evaluation (*val-2017*), respectively. In addition, 20k images in *test-dev* did not have any disclosed labels. We conducted an ablation study and reported the final result for *val-2017* and *test-dev*. Results for MS COCO were reported using mAP, mAP_50_ (mAP scores with IoU of 0.5), and mAP_75_ (mAP scores with IoU of 0.75). Here, mAP_S_, mAP_M_, and mAP_L_ correspond to results on small, medium, and large scales, respectively.

### Implementation details

The NAS-gate convolutional module was designed such that it could be install on all members of the ResNet backbone family, i.e., ResNet-50, ResNet-101, ResNeXt^45^ ,and ResNeSt^46^. However, owing to the limited GPU memory of our hardware environment, we conducted the experiment using only two backbones, i.e., ResNet-50 and ResNet-101. However, if only hardware GPU memory is secured, then expanding the proposed methods to a detector model (e.g., Mask R-CNN and Cascade Mask R-CNN) that requires expensive computational memory will not be an issue as there is no difference in module installation. In our implementation, we replaced all the $3\times 3$ convolutional operations of the ResNet-50 and ResNet-101 backbones with the NAS-gate convolutional module. Furthermore, the capsule attention module was adopted in the highest level of the FPN and PAFPN, as shown in Fig. 1b, d. We implemented our model using MMDetection^47^, an open-source object detection toolbox based on PyTorch. Both of the proposed modules were implemented on two-stage detectors such as Faster R-CNN^28^ and Cascade R-CNN^15^ as well as one-stage detectors such as RetinaNet^6^ and FCOS^7^. In the experiments on PASCAL VOC, the models were trained for four epochs and training was repeated on the training dataset three times per epoch with an initial learning rate of 0.01. The learning rate was multiplied by 0.1 every three epochs. Furthermore, we trained the model on MS COCO for 12 epochs with an initial learning rate of 0.02. After eight and 11 epochs, the learning rate was multiplied by 0.1. We used the SGD optimizer with momentum, that equal to 0.9 to minimize the summation of the cross-entropy loss for classification prediction head and smooth L1 loss with beta=1.0 for bounding box prediction head. In addition, we resized the input images to the same size, i.e., 1333 $\times$ 800, and trained the model with a batch size of four images per GPU on an environment equipped with NVIDIA Titan Xp GPU, CUDA version 10.2, and PyTorch 1.5.

## Results

In order to evaluate the effectiveness of each proposed module, we conducted experiments comparing the existing method with similar concepts or methods by using the same dataset for training and testing, including the same software and hardware environment in each experiment for a fair comparison. Moreover, in Tables 1–6 showing the experimental results, the best value for each metric is highlighted in bold.

### NAS-gate convolutional module

We examined the effectiveness of the proposed NAS-gate convolutional module on MS COCO *test-dev*. We evaluated the performance of the NAS-gate convolutional module incorporated in Cascade R-CNN with FPN and ResNet-101 against existing NAS backbone-based object detectors including DetNAS, AmoebaNet, and Hit-Detector. Table 1 indicates that the Cascade R-CNN with FPN with the proposed NAS-gate convolutional module implemented on ResNet-101 outperforms the other NAS backbones, with a mAP of 43.5%.

**Table 1:** Comparison of the performance of the NAS-gate convolutional module and other NAS backbone frameworks on MS COCO *test-dev*.

Method	Backbone	mAP	mAP_50_	mAP_75_	mAP_S_	mAP_M_	mAP_L_
DetNAS	DetNAS	37.9	60.1	41.2	22.7	41.2	48.3
AmoebaNet w FPN	AmoebaNet	43.4	–	–	–	–	–
Hit-detector	Hit-detector	41.4	**62.4**	45.9	**25.2**	45.0	54.1
Cascade R-CNN w FPN	ResNet-101 (NAS-gate convolutional module)	**43.5**	62.2	**47.3**	24.5	**47.6**	**57.7**

**Table 2:** Comparison performance of capsule attention module with other attention modules on MS COCO *val-2017*.

Method	Backbone	Attention module	mAP	mAP_50_	mAP_75_	mAP_S_	mAP_M_	mAP_L_
Faster R-CNN with FPN	ResNet-50	x	37.4	58.1	40.4	21.2	41.0	48.1
	ResNet-50	CBAM^40^	32.8	53.5	34.5	19.2	35.9	41.9
	ResNet-50	SE Module^39^	32.6	53.8	34.7	19.0	35.4	42.1
	ResNet-50	Capsule Attention	**38.0**	**59.2**	**41.3**	**22.2**	**41.8**	**49.1**
Faster R-CNN with FPN	ResNet-101	x	39.4	60.1	43.1	22.4	43.7	51.1
	ResNet-101	CBAM^40^	39.4	61.1	42.8	23.0	43.6	51.2
	ResNet-101	SE Module^39^	38.9	60.1	42.1	22.3	43.2	51.0
	ResNet-101	Capsule Attention	**39.8**	**60.6**	**43.3**	**23.5**	**43.7**	**52.2**

**Table 3:** Effect of each proposed module on MS COCO *val-2017*.

Method	Backbone	NAS-Gate conv	Capsule attention	mAP	mAP_50_	mAP_75_	mAP_S_	mAP_M_	mAP_L_
Faster R-CNN w FPN	ResNet-50	$\times$	$\times$	37.4	58.1	40.4	21.2	41.0	48.1
	ResNet-50	$\checkmark$	$\times$	39.9 (+ 2.5)	61.1 (+ 3.0)	43.2 (+ 2.8)	22.9 (+ 1.7)	43.3 (+ 2.3)	51.9 (+ 3.8)
	ResNet-50	$\times$	$\checkmark$	38.0 (+ 0.6)	59.2 (+ 1.1)	41.3 (+ 0.9)	22.2 (+ 1.0)	41.8 (+ 1.4)	49.1 (+ 1.0)
	ResNet-50	$\checkmark$	$\checkmark$	**40.1 (+ 2.7)**	**61.2 (+3.1)**	**43.6 (+ 3.2)**	**23.5 (2.3)**	**43.8 (+ 2.8)**	**52.5 (+ 4.4)**
Faster R-CNN w FPN	ResNet-101	$\times$	$\times$	39.4	60.1	43.1	22.4	43.7	51.1
	ResNet-101	$\checkmark$	$\times$	41.1 (+ 1.7)	61.8 (+ 1.7)	44.6 (+ 1.5)	23.6 (+ 1.2)	45.0 (+ 1.3)	54.0 (+ 2.9)
	ResNet-101	$\times$	$\checkmark$	39.8 (+ 0.4)	60.6 (+ 0.5)	43.3 (+ 0.2)	23.5 (+ 1.1)	43.7 (+ 0.0)	52.2 (+ 1.1)
	ResNet-101	$\checkmark$	$\checkmark$	**41.4 (+ 2.0)**	**62.3 (+ 2.2)**	**45.1 (+ 2.0)**	**24.0 (+ 1.6)**	**45.5 (+ 1.8)**	**54.3 (+ 3.2)**

### Capsule attention module

To validate the effectiveness of the proposed capsule attention module, we evaluated the performance of a baseline Faster R-CNN with FPN and two different backbones (ResNet-50 and ResNet-101) with various exist attention mechanisms (CBAM^40^ and SE Module from SENet^39^) including the capsule attention module on MS COCO *val-2017*. Furthermore, the competing attention modules (CBAM and SE module) were adopted at the highest level of FPN, just like the capsule attention module in Fig. 1b. It can be observed from Table 2 that the capsule attention module enhanced the mAP of the baseline detectors on the ResNet-50 and ResNet-101 backbones while outperforming the other attention modules in terms of the mAP.

### Impact of proposed modules on the quantitative performance

To evaluate the impact of the proposed NAS-gate convolutional module and capsule attention module on MS COCO *val-2017*, we compared the box mAP of the baseline detectors (Faster R-CNN with FPN using ResNet-50 and ResNet-101 as a backbone) with the baseline detectors incorporating the proposed modules. As presented in Table 3, adding the NAS-gate convolutional module improved the mAP by 2.5% and 1.7% on the ResNet-50 and ResNet-101 backbone, respectively. Furthermore, adding the capsule attention improved the mAP by 0.6% and 0.4% on the ResNet-50 and ResNet-101 backbone, respectively. Combining the two proposed modules improved the mAP by 2.7% and 2.0% on the ResNet-50 and ResNet-101 backbone, respectively.

**Table 4:** Comparison of the results obtained using the proposed module for MS COCO *test-dev* with different detectors.

Method	Backbone	mAP	mAP_50_	mAP_75_	mAP_S_	mAP_M_	mAP_L_
**One-stage detectors**							
RetinaNet^6^	ResNet-101	39.1	59.1	42.3	21.8	42.7	50.2
RetinaNet	ResNet-101 [NAS-gate convolutional module] + FPN[CapAtt module]	41.1**(+ 2.0)**	60.8 **(+ 1.7)**	44.1 **(+ 1.8)**	23.0 **(+ 1.2)**	44.1 **(+ 1.4)**	52.7 **(+ 2.5)**
FCOS^7^ w GN and w/o MS training	ResNet-101-FPN	39.3	59.1	42.1	22.2	42.3	49.4
FCOS w GN and w/o MS training	ResNet-101 [NAS-gate convolutional module] + FPN[CapAtt module]	41.3 **(+ 2.0)**	60.8 **(+ 1.7)**	44.4 **(+ 2.3)**	23.3 **(+ 1.1)**	44.3 **(+ 2.0)**	52.6 **(+ 3.2)**
**Two stage detectors**							
Faster R-CNN with FPN^17^	ResNet-101	36.2	59.1	39.0	18.2	39.0	48.2
Faster R-CNN with FPN	ResNet-101 [NAS-gate convolutional module] + FPN [CapAtt module]	42.0 **(+ 5.8)**	62.7 (**(+ 3.6)**	45.8**(+6.8)**	23.8 **(+ 5.6)**	45.0 **(+ 6.0)**	53.3 **(+ 5.1)**
Cascade R-CNN^15^	ResNet-101	42.8	62.1	46.3	23.7	45.5	55.2
Cascade R-CNN	ResNet-101 [NAS-gate convolutional module] + PAFPN [CapAtt module]	43.8 **(+ 1.0)**	62.8 **(+ 0.7)**	47.7 **(+ 1.4)**	24.7 **(+ 1.0)**	46.6 **(+ 1.0)**	56.5 **(+ 1.3)**

**Table 5:** State-of-the-art comparison on PASCAL VOC - *VOC-2007 test* for bounding box object detection.

Method	Backbone	mAP_50_
**One-stage detectors**		
SSD512	VGG16	76.8
RetinaNet	ResNet-50	77.3
FCOS	ResNet-101	74.9
**Two-stage detectors**		
Faster R-CNN w FPN	ResNet-50	80.05
Faster R-CNN wFPN	ResNet-101	81.83
Cascade R-CNN w FPN	ResNet-101	81.83
**NAS-based backbone detectors**		
DetNAS	DetNAS	81.5
Auto-FPN	ResNet-50	81.8
**Proposed method**		
NASGC-CapANet [Faster R-CNN w FPN]	ResNet-50	81.96
NASGC-CapANet [Faster R-CNN w FPN]	ResNet-101	82.64
NASGC-CapANet [Faster R-CNN w PAFPN]	ResNet-50	82.36
NASGC-CapANet [Faster R-CNN w PAFPN]	ResNet-101	**82.70**

**Table 6:** State-of-the-art comparison on MS COCO *test-dev* for bounding box object detection.

Method	Backbone	mAP	mAP_50_	mAP_75_	mAP_S_	mAP_M_	mAP_L_
**One-stage detectors**							
SSD512^10^	VGG16	28.8	48.5	30.3	10.9	31.8	43.5
YOLOv3^4^	DarkNet-53	33.0	57.9	34.4	18.3	25.4	41.9
RetinaNet^6^	ResNet-101	39.1	59.1	42.3	21.8	42.7	50.2
RetinaNet^6^	ResNeXt-101	40.8	61.1	44.1	24.1	44.2	51.2
RefineDet512^48^	ResNet-101	36.4	57.4	39.5	16.6	39.9	51.4
CornerNet^2^	Hourglass-104	42.2	57.8	45.2	20.7	44.8	56.6
ExtremeNet^49^	Hourglass-104	43.7	60.5	47.0	24.1	**46.9**	57.6
FCOS^7^	ResNet-101-FPN	41.5	60.7	45	24.4	44.8	51.6
FCOS w GN and w/o MS training^7^	ResNet-101 - FPN	39.3	59.1	42.1	22.2	42.3	49.4
**Two-stage detectors**							
Faster R-CNN^28^	ResNet-101	34.9	55.7	37.4	15.6	38.7	50.9
Faster R-CNN with FPN^17^	ResNet-101	36.2	59.1	39.0	18.2	39.0	48.2
Mask R-CNN with FPN^13^	ResNet-101	38.2	60.3	41.7	20.1	41.1	50.2
Cascade R-CNN^15^	ResNet-101	42.8	62.1	46.3	23.7	45.5	55.2
Cascade mask R-CNN^15^	ResNet-101	43.3	61.7	47.2	24.2	46.3	**58.2**
Libra R-CNN^50^	ResNet-101	40.3	61.3	43.9	22.9	43.1	51.0
HRNet (Faster R-CNN)^31^	HRNetV2p-W32	41.1	62.3	44.9	24.0	43.1	51.4
HRNet (Cascade R-CNN)^31^	HRNetV2p-W32	43.7	62.0	47.4	25.5	46.0	55.3
DetNet^33^	DetNet-59	37.9	60.1	41.2	22.7	41.2	48.3
FishNet^32^	FishNet-150	40.6	–	–	23.3	43.9	53.7
**NAS based backbone detectors**							
DetNAS^51^	DetNAS	37.9	60.1	41.2	22.7	41.2	48.3
AmoebaNet w FPN^52^	AmoebaNet	43.4	–	–	–	–	–
Auto-FPN^37^	ResNet-50	40.5	61.5	43.8	**25.6**	44.9	51.0
Hit-Detector^35^	Hit-Dedector	41.4	62.4	45.9	25.2	45.0	54.1
**Proposed method**							
NASGC-CapANet [RetinaNet]	ResNet-101	41.1	60.8	44.1	23.0	44.1	52.7
NASGC-CapANet [FCOS w GN and w/o MS training]	ResNet-101	41.3	60.8	44.4	23.3	44.3	52.6
NASGC-CapANet [Faster R-CNN w FPN]	ResNet-50	40.3	61.5	43.9	23.1	43.0	50.6
NASGC-CapANet [Faster R-CNN w FPN]	ResNet-101	41.7	62.6	45.4	23.6	44.6	53.0
NASGC-CapANet [Faster R-CNN w PAFPN]	ResNet-101	42.0	62.7	45.8	23.8	45.0	53.3
NASGC-CapANet [Cascade R-CNN w PAFPN]	ResNet-101	**43.8**	**62.8**	**47.7**	24.7	46.6	56.5

To examine the impact of using both of the proposed modules, we implemented the proposed modules on one-stage as well as two-stage detectors. Table 4 presents the performance comparison on MS COCO *test-dev* of the baseline detector and the baseline detector equipped with the proposed modules attached. We tested our modules against two state-of-the-art one-stage object detectors using ResNet-101 as a backbone, i.e., RetinaNet^6^ and FCOS with group normalization and without multi-scale training^7^. The results indicated that using both the proposed modules could enhance the mAP by 2.0%. In the case of two-stage object detectors, we compared the performance of Faster R-CNN and Cascade R-CNN with and without our proposed modules (baseline). As indicated in Table 4, the proposed modules effectively improved the mAP by 5.5% and 1.0% when using the Faster R-CNN and Cascade R-CNN, respectively.

**Figure 4:**
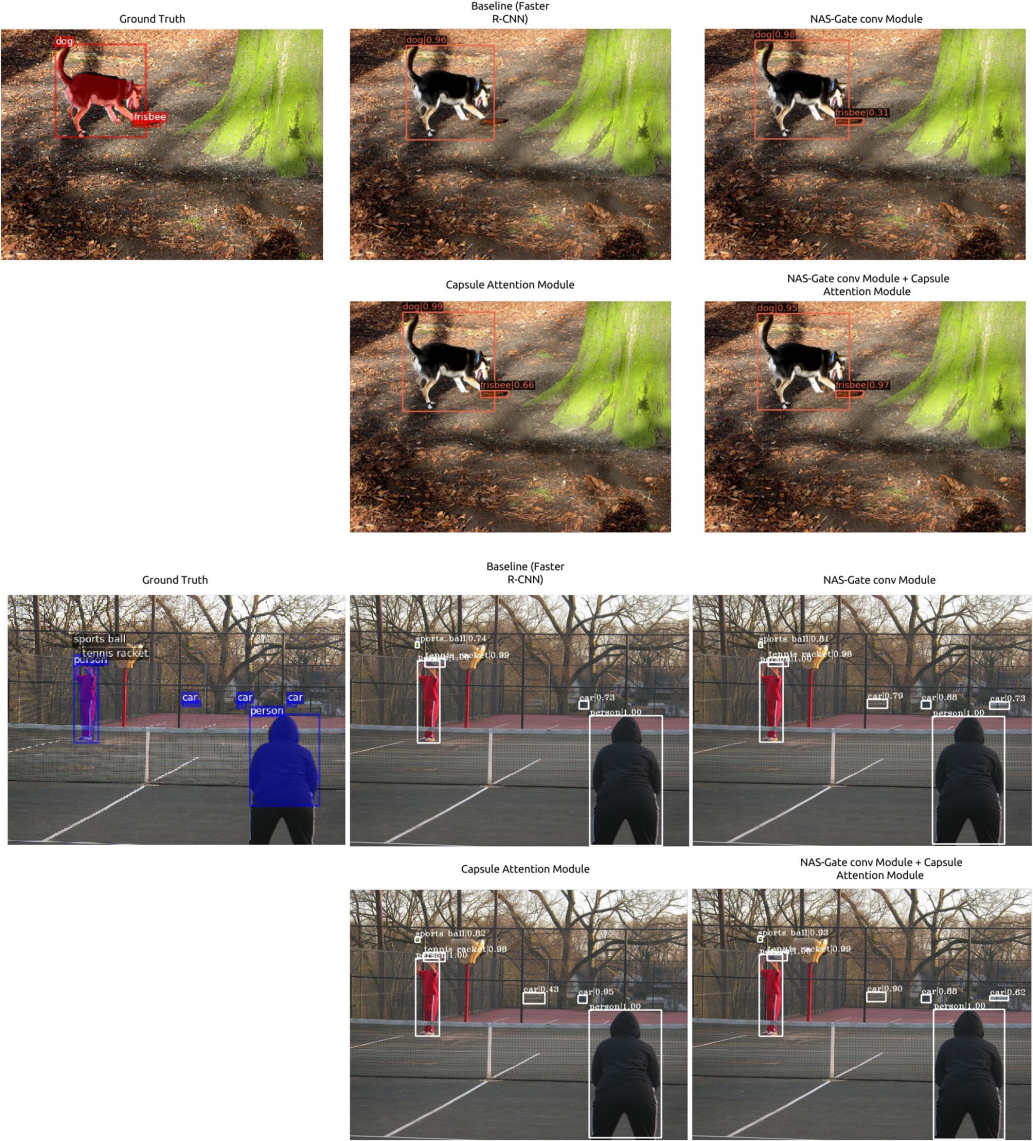
Visualization of the impact of the proposed NAS-gate convolutional module and capsule attention module to detect small object in the image.

**Figure 5:**
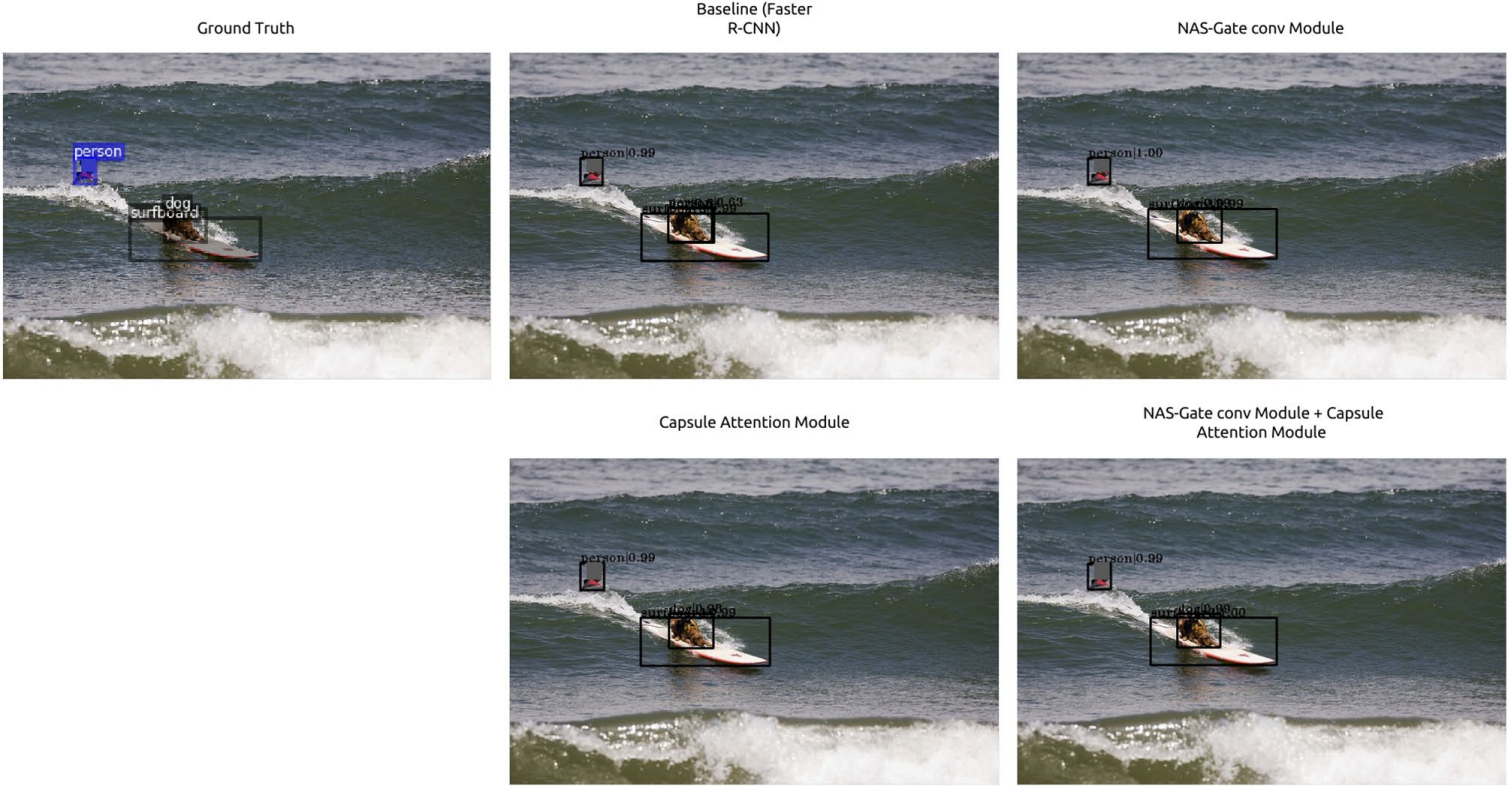
Visualization of the impact of the proposed NAS-gate convolutional module and capsule attention.

### Impact of proposed modules on the qualitative performance

For a qualitative performance analysis of the proposed modules, we visualized the impact of each module, as shown in Figs. 4, 5. The result of the baseline Faster R-CNN is listed in the upper row in Fig. 4; the baseline model could not detect the frisbee that is the small object and the overlapping bounding box with the dog bounding box. In contrast, the proposed modules could successfully detect the small bottle with a high probability. In another case, as presented in the bottom rows in Fig. 4, the baseline Faster R-CNN could detect only one car from three small cars located in the background. In contrast, when only the NAS-gate convolutional module or capsule attention module was incorporated in the baseline detector, the model could successfully recognize more small cars located in the background with higher confidence than the baseline detector. As presented in Fig. 4, besides improving the performance of detecting small objects in the images, the use of the proposed modules led to an enhanced detector localization performance and could predict high-quality bounding boxes that could precisely cover the objects. As listed in the top row in Fig. 4, the baseline Faster R-CNN erroneously recognized one dog as two different objects (dog and human). However, after adding only one of our proposed two different modules, the detector correctly detected the object as a single object. When both the modules were used in the baseline, the bounding box was set up correctly with higher confidence. Thus, it can be inferred that the capsule attention module and NAS-gate convolutional module can alleviate the scale variance problem and enhance object localization in the image.

### Comparisons with state-of-the-art detectors

The main results of NASGC-CapANet are summarized in Tables 5 and 6. We compared the performance of the proposed module with that of state-of-the-art detectors on MS COCO *test-dev* and PASCAL VOC - *VOC-2007*. The performances of state-of-the-art detectors were obtained from the original research experiment results of each method. To compare the performance on the PASCAL VOC dataset, we implemented the proposed modules on state-of-the-art detectors, namely, Faster R-CNN with FPN and Faster R-CNN with PAFPN. Similarly, to evaluate the performance on MS COCO, we used Faster R-CNN with FPN, Faster R-CNN with PAFPN, and Cascade R-CNN with PAFPN. All the models utilized either ResNet-50 or ResNet-101 as a backbone. The presented results are divided into four categories, i.e., one-stage detectors; two-stage detectors; NAS-based backbone detectors, which are similar to the proposed backbone; and the proposed approach, NASGC-CapANet. As summarized in Tables 5 and 6, the proposed NASGC-CapANet achieves an mAP_50_ of 82.70% and 43.8% on PASCAL VOC -*VOC-2007* and MS COCO *test-dev*, respectively.

## Discussion

In this study, we proposed a new object detector, NASGC-CapANet, which combines the state-of-the-art object detector with two newly proposed modules: a NAS-gate convolutional module and capsule attention module. The NAS-gate convolutional module replaces the standard convolutional operation of the classification network backbones and is designed to enhance the feature extraction ability of the backbone network using the NAS gradient method. It utilizes various conditions of convolutional operations such as different kernel sizes and dilated rates of convolution in order to improve the performance of the detector in recognizing objects of varied scales in the images. Furthermore, the NAS-gate convolutional module can optimize the object detector’s backbone architecture with lower computation cost compared to existing NAS-based object detectors. We also introduced a new concept for the attention mechanism, called capsule attention module. The capsule attention module utilizes the global context to improve feature representation by concentrating on object-relevant features without losing spatial relationships. We adopt the capsule attention module in FPN and FPN-based methods in order to mitigate the information loss at the highest level of the FPN and FPN-based methods as well as enhance the localization of the detectors.

We conducted an experiment to evaluate the performance of both proposed modules and NASGC-CapANet on some public object detection datasets, i.e., PASCAL VOC and MS COCO. The experimental results show that replacing the convolutional operation of the ResNet-50 and ResNet-101 backbone outperforms the NAS-based object detectors’ backbone. In addition, adopting capsule attention module at the highest level of FPN improves upon the performance of the existing attention mechanism. Furthermore, NASGC-CapANet, which combines both proposed modules with state-of-the-art object detectors, can significantly outperform baseline detectors. NASGC-CapANet-based Faster-RCNN has a 5.8% higher mAP than the baseline Faster-RCNN on MS COCO *test-dev*. We also analyzed the qualitative performance of NASGC-CapANet with the baseline object detector. The results demonstrate that the detection performance of NASGC-CapANet is more accurate in terms of multiscale object recognition and localization.

## Data Availability

The source code for our model located at https://github.com/Ewha-AI/Object-Detection_COCO.git.
